# Treatment Outcomes of Patients with Locally Advanced Synchronous Esophageal and Head/Neck Squamous Cell Carcinoma Receiving Curative Concurrent Chemoradiotherapy

**DOI:** 10.1038/srep41785

**Published:** 2017-01-30

**Authors:** Yen-Hao Chen, Hung-I. Lu, Chih-Yen Chien, Chien-Ming Lo, Yu-Ming Wang, Shang-Yu Chou, Yan-Ye Su, Li-Hsueh Shih, Shau-Hsuan Li

**Affiliations:** 1 Department of Hematology-Oncology, Kaohsiung Chang Gung Memorial Hospital and Chang Gung University College of Medicine, Kaohsiung, Taiwan; 2 Graduate Institute of Clinical Medical Sciences, Chang Gung University College of Medicine, Taiwan; 3 Department of Thoracic & Cardiovascular Surgery, Kaohsiung Chang Gung Memorial Hospital and Chang Gung University College of Medicine, Kaohsiung, Taiwan; 4 Department of Otolaryngology, Kaohsiung Chang Gung Memorial Hospital and Chang Gung University College of Medicine, Kaohsiung, Taiwan; 5 Department of Radiation Oncology, Kaohsiung Chang Gung Memorial Hospital and Chang Gung University College of Medicine, Kaohsiung, Taiwan; 6 Department of Nursing, Kaohsiung Chang Gung Memorial Hospital, Kaohsiung, Taiwan

## Abstract

The present study investigated clinical outcomes and prognostic factors of patients with locally advanced synchronous esophageal squamous cell carcinoma (ESCC) and head/neck squamous cell carcinoma (HNSCC) receiving curative concurrent chemoradiotherapy (CCRT), and determined whether synchronous ESCC/HNSCC patients had worse prognosis compared to isolated ESCC patients. Using propensity score matching method, we compared 60 locally advanced synchronous ESCC/HNSCC patients with 60 matched isolated ESCC patients. Compared to 60 matched isolated ESCC patients, synchronous ESCC/HNSCC patients had significantly worse prognosis (13.5 months versus 17.2 months, P = 0.01), more grade 3–4 CCRT toxicity, and higher percentage of CCRT interruption. For synchronous ESCC/HNSCC group, the 1-year and 2-year survival rates were 52% and 13%, respectively. Univariate analysis showed that early ESCC stage, non-T4b disease, and salvage operations were significantly associated with superior survival. In multivariate analysis, ESCC stage represented an independent prognosticator. For chemotherapy regimen during CCRT, cisplatin/5-fluorouracil had significantly more grade 3–4 mucositis/esophagitis and neutropenia than weekly cisplatin. In conclusion, synchronous ESCC/HNSCC patients receiving curative CCRT have worse prognosis and poorer compliance of CCRT compared to isolated ESCC patients. For these patients, ESCC stage and T4b disease were significantly associated with clinical outcomes, and salvage operation may improve overall survival.

Esophageal cancer and head/neck cancer are among the most frequently occurring malignancies worldwide. In several Eastern and Asian countries, esophageal cancer and head/neck cancer are very common, and approximately 90% of these cancers are squamous cell carcinomas^1^,^2^. The risk factors of esophageal squamous cell carcinoma (ESCC) and head/neck squamous cell carcinoma (HNSCC) include long-term use of tobacco and alcohol, betel quid chewing, chronic mucosal irritation, and upper aerodigestive cancer history^3^,^4^. The term “field cancerization”, meaning multifocal synchronous and metachronous carcinogenesis in the upper aerodigestive tract, was coined by Slaughter in 1953^5^,^6^. In Taiwan, 15–20% of patients with HNSCC may develop a secondary ESCC, and vice versa. In patients with a new diagnosis of ESCC, routine screening of head and neck field is necessary and results in more frequent detection of second primary HNSCC. On the other hand, patients diagnosed with HNSCC receive routine endoscopy of the esophagus to exclude second primary ESCC. The treatment guidelines for ESCC and HNSCC have been well documented, but the clinical course and management of synchronous ESCC/HNSCC remain unclear. The location, extent of tumor invasion, and anatomic proximity of each cancer complicate the therapeutic strategy and limit treatment options^7^. In the past, these patients could only undergo a surgical resection of the synchronous ESCC/HNSCC, but the clinical outcomes of this treatment were very poor and the chance of cure was very small^8^. Several studies focusing on surgical treatment for synchronous ESCC/HNSCC patients have been reported^9^,^10^. The causes of their typically poor prognosis were found to be related to the difficulty of operation, higher rates of complications, patient intolerance, and disease progression, such that these patients were generally thought to be candidates for palliative care^8^. Over time, however, significant improvements have been made in chemoradiotherapy, yielding another treatment option for these patients.

In clinical practice, curative concurrent chemoradiotherapy (CCRT) is often used to treat patients with non-metastatic synchronous ESCC/HNSCC. If either the ESCC or the HNSCC of the synchronous ESCC/HNSCC is in a locally advanced stage, CCRT rather than surgical resection is preferred. However, to the best of our knowledge, there have been very few studies that have investigated the clinical outcomes and prognostic factors of curative CCRT for such patients. In the present study, we retrospectively analyzed locally advanced ESCC patients who underwent CCRT as curative treatment in our hospital. Among these patients, locally advanced synchronous ESCC/HNSCC patients were also identified. The aim of our study was to evaluate the clinical outcomes and prognostic factors of locally advanced synchronous ESCC/HNSCC patients receiving curative CCRT, and to determine whether locally advanced synchronous ESCC/HNSCC patients had worse prognoses compared to isolated ESCC patients.

## Results

### Comparison between isolated ESCC and locally advanced synchronous ESCC/HNSCC

We retrospectively reviewed our ESCC database, and 692 ESCC patients who received curative CCRT were identified. Of the 60 locally advanced synchronous ESCC/HNSCC patients, all the patients were men and had a mean age of 52 years (range: 35 to 71 years). Fifty-three patients (88%) had history of tobacco smoking and alcohol consumption were mentioned in 50 patients (83%). The tumor stages for each patient were defined according to the AJCC 7^th^ staging system. For the ESCC cancer, 9 patients (15%) were found to have a stage I tumor, 10 patients (17%) were found to have a stage II tumor, and 41 patients (68%) were found to have a stage III tumor, while for the HNSCC patients, 4 patients (7%) were found to have a stage I tumor, 4 patients (7%) were found to have stage II tumor, 8 patients (13%) were found to have a stage III tumor, and 44 patients (73%) were found to have a stage IVA or stage IVB tumor. The primary tumor location for the ESCC was found to be the upper esophagus in 18 patients (30%), the middle esophagus in 21 patients (35%), and the lower esophagus in 21 patients (35%). The grade of ESCC were found to be grade 1 in 17 patients (28%), grade 2 in 35 patients (59%), and grade 3 in 8 patients (13%); for HNSCC patients, the grade 1, 2, and 3 were mentioned in 20 patients (33%), 31 patients (52%), and 9 patients (15%), respectively. The origin of the HNSCC included the oropharynx in 12 patients (20%), the hypopharynx in 39 patients (65%), and the larynx in 9 patients (15%).

**Figure 1:**
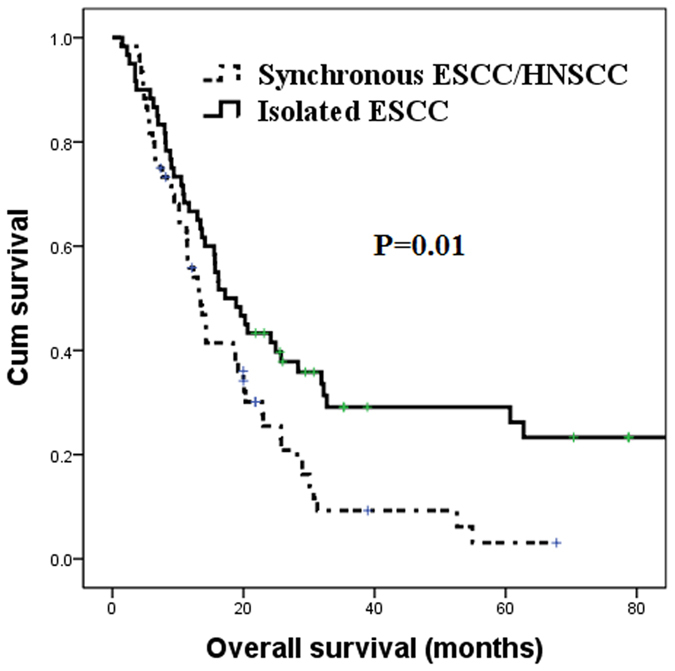
The overall survival curves of 60 patients with locally advanced synchronous ESCC/HNSCC compared to 60 matched patients with isolated ESCC^#^. ^#^Patients were matched using the propensity score matching method. ESCC: esophageal squamous cell carcinoma; HNSCC: head and neck squamous cell carcinoma.

Among the 632 isolated ESCC patients, 60 matched patients were identified for comparison with the synchronous group using the propensity score matching method. Age, sex, ESCC stage, and ESCC location were all matched so that there was no statistical difference between these two groups. Patients with isolated ESCC were found to have superior survival over those with locally advanced synchronous ESCC/HNSCC (17.2 months versus 13.5 months, P = 0.01, Fig. 1). The clinicopathological parameters of these patients are shown in Tables 1 and 2.

### Clinical outcomes of patients with locally advanced synchronous ESCC/HNSCC receiving curative CCRT

**Figure 2:**
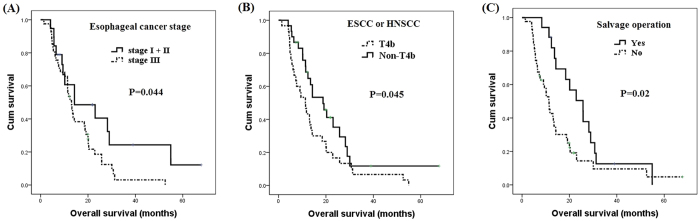
Comparison of survival curves of 60 patients with locally advanced synchronous esophageal and head/neck squamous cell carcinoma according to clinical features. (**A**) Esophageal cancer stage (**B**) T4b disease status (**C**) Salvage operation.

The 1-year and 2-year survival rates of these patients were 52% and 13%, respectively. Cross tabulation of tumor location and cancer stage was shown in Table 3, and the characteristics and survival outcomes of the locally advanced synchronous ESCC/HNSCC patients are shown in Table 4. According to univariate analysis, there were no significant differences in overall survival in terms of age, history of tobacco smoking and alcohol consumption, tumor location of the ESCC and HNSCC, or tumor grade of ESCC and HNSCC. A total of 19 early ESCC stage I and stage II patients had significantly superior overall survival compared to 41 locally advanced ESCC stage III disease patients (14.3 months versus 13.0 months, P = 0.044, Fig. 2A). The 16 patients who had HNSCC stage I, stage II or stage III disease had better overall survival than 44 HNSCC stage IVA and stage IVB patients, but the statistical difference only reached borderline significance. In all, 30 patients had T4b disease, whether ESCC or HNSCC, and these patients had worse overall survival than the rest of the patients without T4b disease (11.3 months versus 18.8 months, P = 0.045, Fig. 2B). There were some patients who received a salvage operation after undergoing CCRT, including 9 patients for ESCC, 10 patients for HNSCC, and a total of 17 patients for ESCC or HNSCC (2 patients underwent a salvage operation for ESCC and HNSCC simultaneously). The patients who underwent a salvage operation for ESCC or HNSCC had superior overall survival in comparison with those who did not undergo a salvage operation (25.7 months versus 11.4 months, P = 0.02, Fig. 2C).

According to multivariate comparison, only early stage ESCC (P = 0.039, hazard ratio: 0.50, 95% confidence interval: 0.26–0.96) represented the independent predictive factors of superior overall survival.

### Adverse events among patients with isolated ESCC versus those with locally advanced synchronous ESCC/HNSCC

In the present study, we found that the patients with locally advanced synchronous ESCC/HNSCC who received curative CCRT had worse prognoses than those with isolated ESCC who received curative CCRT. Therefore, we also evaluated if there were any differences in terms of compliance with or adverse events associated with CCRT between these two groups. The adverse events among these patients are shown in Table 5. There was a higher percentage of CCRT interruptions lasting more than one week among the synchronous group than among the isolated group (18% versus 7%, P = 0.048). The incidence of radiation mucositis/esophagitis (100% versus 98%) and radiation dermatitis (100% versus 100%) were similar between these two groups, but the synchronous group had more grade 3–4 radiation mucositis/esophagitis (33% versus 15%, P = 0.032) and radiation dermatitis (28% versus 12%, P = 0.039) compared to the isolated group. In addition, the patients with locally advanced synchronous ESCC/HNSCC suffered from more hematologic toxicities than those with isolated ESCC, including neutropenia (63% versus 43%, P = 0.022), anemia (92% versus 78%, P = 0.036), and thrombocytopenia (68% versus 52%, P = 0.047). Severe adverse events with grade 3–4 neutropenia were also more predominant among the synchronous group than among the isolated group (28% versus 13%, P = 0.035). The synchronous group also had a higher percentage of grade 3–4 thrombocytopenia, but the statistical difference between the two groups was not significant.

Some patients were admitted to the hospital due to treatment-related complications, such as pneumonia, dysphagia, tumor bleeding, upper gastrointestinal bleeding, etc. There were more patients admitted for complications in the synchronous group than in the isolated group (38% versus 22%, P = 0.036).

### Adverse events among locally advanced synchronous ESCC/HNSCC patients receiving different chemotherapy regimens

According to different chemotherapy regimens, all 60 locally advanced synchronous ESCC/HNSCC patients were divided into two groups: 49 patients received cisplatin/5-fluorouracil, and 11 patients underwent weekly cisplatin. CCRT interruptions lasting more than one week, grade 3–4 radiation dermatitis, and grade 3–4 thrombocytopenia in the cisplatin/5-fluorouracil group were higher than those in the weekly cisplatin group, but the statistical difference was not significant. Compared to the patients in the weekly cisplatin group, patients in the cisplatin/5-fluorouracil group had significantly higher percentage of grade 3–4 neutropenia (35% versus 0%, P = 0.021). In addition, patients in the cisplatin/5-fluorouracil group had higher grade 3–4 radiation mucositis/esophagitis compared with patients in the weekly cisplatin group, but it only reached a marginal trend toward significance (39% versus 9%, P = 0.059). The adverse events among these patients are shown in Table 6.

## Discussion

Locally advanced synchronous ESCC/HNSCC is a small population of patients diagnosed with ESCC or HNSCC, and the large field of tumor involvement limits the efficacy and feasibility of treatment options. To the best of our knowledge, there have been only a few studies that have evaluated and discussed the efficacy and feasibility of the various treatment options. In the past, surgical resection of the tumors was regarded as a standard treatment^11^,^12^. Some previous studies showed that the mean survival time for patients with synchronous esophageal and head/neck cancer was similar to that of isolated esophageal cancer, but the feasibility of surgery is related to many factors, such as the severity of tumor invasion, the performance status, co-morbidities, etc. Recently, some studies have reported the clinical outcomes of synchronous esophageal and head/neck cancer patients treated with chemotherapy, radiotherapy, surgery, or combination therapy^13^,^14^,^15^. Miyazato *et al*. reported that patients with double cancers had a worse 5-year survival rate than those with isolated esophageal cancer^16^. In our study, we found that the locally advanced synchronous ESCC/HNSCC patients had worse prognoses than the isolated ESCC patients. In addition, we found that patients with locally advanced synchronous ESCC/HNSCC had poorer compliance with CCRT and experienced more adverse events during CCRT treatment, both of which at least partially contributed to the worse prognoses for these patients. We suggest that the large field of radiotherapy for synchronous double cancers may cause more adverse events, leading to interruptions of treatment.

The locally advanced synchronous ESCC/HNSCC patients receiving CCRT were subjected, on average, to a larger field of radiotherapy than the isolated ESCC or HNSCC patients, so high grade radiation mucositis/esophagitis, dermatitis, and bone marrow suppression were leading complications, all of which may cause treatment interruption. More than 60% of the patients had hematologic toxicity, including neutropenia, anemia, and thrombocytopenia in our study. Due to the aforementioned reasons, nearly 40% of the patients were admitted to the hospital for CCRT-related complications, such as pneumonia, gastrointestinal bleeding, tumor bleeding, or other clinical conditions.

Our study revealed esophageal cancer stage was more important in predicting outcome of synchronous ESCC/HNSCC patients. Shinoto *et al*. also reported that the outcomes of synchronous ESCC/HNSCC patients were significantly affected by esophageal cancer stage^17^. Although head/neck cancer and esophageal cancer were both squamous cell carcinoma and had the same risk factors, their treatment outcomes are different. According to the 7th American Joint Committee on Cancer (AJCC) staging system^18^, the 5-year overall survival rates of stage III and stage IV hypopharyngeal cancer were 36% and 24%, respectively. The 5-year overall survival rates of stage III and stage IV glottis cancer were 56% and 44%, respectively. The 5-year overall survival rates of stage III and stage IV supraglottis cancer were 53% and 34%, respectively. The 5-year overall survival rate of stage IV oropharyngeal cancer was around 30~40%. However, the 5-year overall survival rates of stage IIIA, IIIB, and IIIC esophageal squamous cell carcinoma were only around 15~25%. Hence, the prognosis of esophageal cancer was worse than that of head and neck cancer which may explain why esophageal cancer stage was more important in predicting outcome of synchronous ESCC/HNSCC patients. This phenomenon may be related to several factors. First, the response rate to induction chemotherapy in HNSCC seems higher than that in ESCC. Previous studies reported the response rates of induction chemotherapy with cisplatin/5-fluorouracil were around 55~70% in patients with HNSCC^19^,^20^,^21^ and 35–45% in patients with ESCC^22^,^23^,^24^. Second, the limitation of radiotherapy dose in HNSCC and ESCC was not quite the same. According to National Comprehensive Cancer Network Guidelines, the suggestive radiotherapy dose for HNSCC was 66–74 Gy, but the dose of radiotherapy delivered to ESCC patients was only 50–50.4 Gy^25^,^26^. The different radiotherapy doses may result in different treatment outcomes. Third, the surgical mortality of ESCC seems higher than those of HNSCC. For example, previous reports revealed that surgical mortality rates were around 4~10% for ESCC patients receiving esophagectomy^27^ and 0.5% for HNSCC patients receiving total laryngectomy^28^.

For either HNSCC or ESCC, T4b status indicates an unresectable disease. Despite advances in surgical technique, surgery alone has not been found to improve the prognoses of patients with T4 esophageal cancers^29^,^30^,^31^. Although downstaging may develop and result in the possibility of surgical resection after definitive CCRT treatment, most patients with T4b disease do not ultimately receive an operation. In our study, one half of the patients had T4b disease status and the other half did not, and the patients with T4b disease had worse prognoses than those without T4b disease.

Although the outcome of the synchronous ESCC/HNSCC patients was poor, some patients still achieved longer survival than others. We reported that 2-year surveil rate was only 13% for these patients, so we selected patients whose survival time more than 2 years, and a total of 11 patients were mentioned in our study. Among the 11 patients, one patient had both stage I ESCC and HNSCC, another one patient had both stage II ESCC and HNSCC, and the rest 9 patients had at least stage III disease. Early ESCC stage is a predictive makers of better prognosis (P = 0.044) and early HNSCC stage had a favorable trend toward prolonged survival (P = 0.062) in our study. On the other hand, 8 (73%) of 11 patients received salvage operation after CCRT. In our analysis, patient underwent salvage operation also had better overall survival than those without surgery (25.7 months versus 11.4 months, P = 0.02). Previous studies also showed CCRT followed by surgery improved overall survival and salvage surgery may be helpful in carefully selected locally advanced ESCC and HNSCC patients^32^,^33^. Therefore, we suggest that early stage and salvage operation may contribute to the prolonged survival.

Our study had several limitations. First, it was a retrospective study of patients treated at a single institution, and the sample size was small. Second, the characteristics of the enrolled patients were very heterogeneous because this disease entity is uncommon. However, to the best of our knowledge, this study, at present, covers the largest series of locally advanced synchronous ESCC/HNSCC patients who underwent curative CCRT and may thus be useful for understanding this rare disease entity.

In conclusion, the results of our study suggest that the treatment outcomes of locally advanced synchronous ESCC/HNSCC patients were worse than isolated ESCC patients, including worse prognoses and higher percentage of CCRT interruptions, hematologic toxicities, and complications-related admissions. For locally advanced synchronous ESCC/HNSCC patients receiving curative CCRT, the esophageal cancer stage and T4b disease status are significantly associated with clinical outcomes, and salvage operations may improve overall survival.

## Methods

### Patient selection

The definition of “synchronous” in our study is that the date of diagnosis of ESCC and HNSCC were within 6 months, and these patients must receive CCRT as initial treatment for double cancers at the same time. First, 1,219 patients with ESCC who were treated at Kaohsiung Chang Gung Memorial Hospital between January 1996 and December 2013 were retrospectively reviewed. Of these 1,219 ESCC patients, we excluded those patients with metastatic disease or those who had a history of malignancy prior to first being treated for the ESCC. After that, only ESCC patients who received CCRT as a curative treatment were included. Finally, a total of 692 ESCC patients were selected. Of these patients, 60 ESCC patients who also had synchronous HNSCC were identified. So these 692 patients were divided into a group of 60 patients with locally advanced synchronous ESCC/HNSCC and a group of 632 patients with isolated ESCC. The majority of these 692 patients had locally advanced status, and they all received CCRT as curative treatment. Any patients who underwent other therapeutic protocols, such as surgical resection of both cancers, surgical resection followed by chemotherapy/radiotherapy, chemotherapy/radiotherapy to treat one cancer and surgical resection of the other cancer, or supportive care, were excluded.

Among the 632 isolated ESCC patients, the propensity score matching method was used to prevent selection bias. We used binary logistic regression to calculate a propensity score, and the covariates entered in the propensity model were age, sex, tumor stage, and tumor location. Subsequently, a 1-to-1 match between patients with locally advanced synchronous ESCC/HNSCC and those with isolated ESCC was obtained using the closest matching score. Finally, 60 matched isolated ESCC patients were selected and considered as a control group. The algorithm used is shown in Supplement Fig. S1.

The pathologies of all the cases of esophageal cancer and head/neck cancer were squamous cell carcinoma. The tumor stages were determined according to the 7^th^ American Joint Committee on Cancer (AJCC) staging system.

### Concurrent chemoradiotherapy planning

In all patients, CT-based radiation therapy treatment planning, immobilization with thermoplastic head-neck-shoulder cast, and linear accelerator to deliver 6-MV or 10-MV photons with a daily dose of 1.8–2 Gy and 5 fractions per week were used. For ESCC, the gross tumor volume (GTV) was defined as the primary tumor and the metastasis lymph nodes revealed in diagnostic chest CT image or PET-CT. The clinical target volume (CTV) included bilateral supraclavicular fossa (SCF), mediastinum, celiac trunk area and esophagus, which depend on the primary tumor location. According to the radiation therapy guideline in our department, CTV of upper third thoracic ESCC may include bilateral SCF, mediastinum and esophagus only. However, for middle third and lower third thoracic ESCC, additional celiac trunk area will also be irradiated. The dose to the CTV with 0.5–1 cm margin, which depend on image-guided radiation therapy (IGRT) or not, in all direction for prophylactic irradiation was between 36–50 Gy. 50–50.4 Gy was delivered to GTV with tumor plus 3–5 cm cephalad and caudal margin and metastatic lymph nodes plus 0.5–1 cm margin. For HNSCC, the GTV was also defined as the primary tumor and the metastasis lymph nodes noted in diagnostic head and neck CT image data or PET-CT. The CTV included risky head and neck area, such as bilateral neck, retropharyngeal lymph node region, oral cavity, larynx, or pharynx, which depend on tumor location and the clinician’s decision. The prophylactic dose to CTV with 0.3–0.5 cm margin, which depend on IGRT or not, was 50–56 Gy for low risk area and 60–66 Gy for high risk area. To the GTV with 0.3–0.5 cm margin, 70 Gy was irradiated. Due to the very large field requiring treated and the complexity of the target volume, whole field intensity-modulated radiotherapy (IMRT) was used for all these patients and we irradiated the head and neck region and esophageal region simultaneously with continuous RT field.

Chemotherapy was performed concurrently with radiotherapy, and consisted of cisplatin (75 mg/m^2^; 4-hour drip) on day 1 and 5-fluorouracil (1000 mg/m^2^; continuous infusion) on days 1–4 every 4 weeks. Some patients received weekly cisplatin (40 mg/m^2^) as another choice of chemotherapy regimen. Carboplatin was prescribed instead of cisplatin for patients with creatinine clearance <60 mL/min.

### Statistical analysis

Statistical analyses were performed using the SPSS 17 software package (IBM, Armonk, NY). The chi-square test, Fisher’s exact test, and *t*-test were used to compare data between the two groups. Overall survival (OS) was calculated from whichever was earlier, the date of diagnosis for the esophageal cancer or the date of diagnosis for the head/neck cancer, to the date of death as a result of all causes or to the date of the last follow-up.

The Kaplan–Meier method was used for univariate survival analysis, and the difference between survival curves was tested by a log-rank test. In a stepwise forward fashion, parameters with P values < 0.05 at the univariate level were entered into a Cox regression model to analyze their relative prognostic importance. For all analyses, two-sided tests of significance were used, with P < 0.05 considered significant.

### Ethics statement

The retrospective analysis was approved by the Chang Gung Medical Foundation Institutional Review Board (104-8838B). All the methods were carried out in accordance with the approved guideline and written informed consent of the patients or their family was not judged necessary for this kind of retrospective study by the Chang Gung Medical Foundation Institutional Review Board.

## Additional Information

**How to cite this article**: Chen, Y.-H. *et al*. Treatment Outcomes of Patients with Locally Advanced Synchronous Esophageal and Head/Neck Squamous Cell Carcinoma Receiving Curative Concurrent Chemoradiotherapy. *Sci. Rep.* **7**, 41785; doi: 10.1038/srep41785 (2017).

**Publisher's note:** Springer Nature remains neutral with regard to jurisdictional claims in published maps and institutional affiliations.

## Supplementary Material

Supplementary Information

## Figures and Tables

**Table 1:** Clinicopathological parameters in locally advanced synchronous ESCC/HNSCC group and isolated ESCC group.

	Synchronous ESCC/HNSCC (N = 60)	^#^Matched isolated ESCC (N = 60)	P value
Age (years)	52	52	1.0
Sex			
Male	60 (100%)	60 (100%)	1.0
Location			
Upper	18 (30%)	18 (30%)	1.0
Middle	21 (35%)	21 (35%)	
Lower	21 (35%)	21 (35%)	
Stage			
I	9 (15%)	9 (15%)	1.0
II	10 (17%)	10 (17%)	
III	41 (68%)	41 (68%)	
Median overall survival (months)	13.5	17.2	0.01*

ESCC: esophageal squamous cell carcinoma; HNSCC: head/neck squamous cell carcinoma.

^#^Using propensity score matching method.

^*^Statistically significant.

**Table 2:** Clinicopathological parameters in 60 locally advanced synchronous ESCC/HNSCC patients.

Characteristics	
Age	52 years old (35–71)
Sex	
Male	60 (100%)
Tobacco smoking	
Yes	53 (88%)
No	7 (12%)
Alcohol consumption	
Yes	50 (83%)
No	10 (17%)
ESCC stage	
I	9 (15%)
II	10 (17%)
III	41 (68%)
ESCC location	
Upper	18 (30%)
Middle	21 (35%)
Lower	21 (35%)
ESCC grade	
1	17 (28%)
2	35 (59%)
3	8 (13%)
HNSCC stage	
I	4 (7%)
II	4 (7%)
III	8 (13%)
IVA + IVB	44 (73%)
HNSCC	
Oropharynx	12 (20%)
Hypopharynx	39 (65%)
Larynx	9 (15%)
HNSCC grade	
1	20 (33%)
2	31 (52%)
3	9 (15%)

ESCC: esophageal squamous cell carcinoma; HNSCC: head/neck squamous cell carcinoma.

**Table 3:** Cross tabulation of tumor location and cancer stage in 60 locally advanced synchronous ESCC/HNSCC patients.

Location	ESCC Upper	ESCC Middle	ESCC Lower
HNSCC Oropharynx	3	4	5
HNSCC Hypopharynx	13	14	12
HNSCC Larynx	2	3	4
**Stage**	**ESCC stage I**	**ESCC stage II**	**ESCC stage III**
HNSCC stage I	1	0	3
HNSCC stage II	1	1	2
HNSCC stage III	2	0	6
HNSCC stage IVA + IVB	5	9	30

ESCC: esophageal squamous cell carcinoma; HNSCC: head/neck squamous cell carcinoma.

**Table 4:** Univariate analysis of overall survival in 60 locally advanced synchronous ESCC/HNSCC patients.

Characteristics	No. of patients	Median OS (months)	P value
Age			
<52 years	28	13.0	0.82
≥52 years	32	13.7	
Tobacco smoking			
Yes	53	13.2	0.93
No	7	13.5	
Alcohol consumption			
Yes	50	13.2	0.24
No	10	20.1	
ESCC stage			
I + II	19	14.3	0.044*
III	41	13.0	
ESCC location			
Upper	18	13.7	0.93
Middle + Lower	42	12.4	
ESCC grade			
1	17	11.8	0.38
2 + 3	43	13.7	
HNSCC stage			
I + II + III	16	23.0	0.062
IVA + IVB	44	11.5	
HNSCC location			
Oropharynx + Larynx	21	18.8	0.42
Hypopharynx	39	11.8	
HNSCC grade			
1	20	13.2	0.38
2 + 3	40	13.7	
ESCC or HNSCC T4b			
Yes	30	11.3	0.045*
No	30	18.8	
Salvage operation			
Yes	17	25.7	0.02*
No	43	11.4	

ESCC: esophageal squamous cell carcinoma; HNSCC: head/neck squamous cell carcinoma; OS: overall survival.

^*^Statistically significant.

**Table 5:** The adverse events of CCRT in locally advanced synchronous ESCC/HNSCC group and isolated ESCC group.

Complications	Synchronous ESCC/HNSCC (N = 60)	^#^Matched isolated ESCC (N = 60)	P value
CCRT interruption > 1 week	11 (18%)	4 (7%)	0.048*
Complications related admission	23 (38%)	13 (22%)	0.036*
Radiation mucositis/esophagitis			
All grade	60 (100%)	59 (98%)	1.0
Grade 3–4	20 (33%)	9 (15%)	0.032*
Radiation dermatitis			
All grade	60 (100%)	60 (100%)	1.0
Grade 3–4	17 (28%)	7 (12%)	0.039*
Neutropenia			
All grade	38 (63%)	26 (43%)	0.022*
Grade 3–4	17 (28%)	8 (13%)	0.035*
Anemia			
All grade	55 (92%)	47 (78%)	0.036*
Grade 3–4	16 (27%)	12 (20%)	0.26
Thrombocytopenia			
All grade	41 (68%)	31 (52%)	0.047*
Grade 3–4	9 (15%)	4 (7%)	0.12

ESCC: esophageal squamous cell carcinoma; HNSCC: head/neck squamous cell carcinoma; CCRT: concurrent chemoradiotherapy.

^#^Using propensity score matching method.

^*^Statistically significant.

**Table 6:** The comparison of adverse events of CCRT in locally advanced synchronous ESCC/HNSCC patients receiving different chemotherapy regimens.

Complications	Cisplatin/5-fluorouracil (N = 49)	Weekly cisplatin (N = 11)	P value
CCRT interruption > 1 week	10 (20%)	1 (9%)	0.38
Complications related admission	19 (39%)	4 (36%)	0.88
Radiation mucositis/esophagitis			
All grade	49 (100%)	11 (100%)	1.0
Grade 3–4	19 (39%)	1 (9%)	0.059
Radiation dermatitis			
All grade	49 (100%)	11 (100%)	1.0
Grade 3–4	16 (33%)	1 (9%)	0.12
Neutropenia			
All grade	33 (67%)	5 (45%)	0.17
Grade 3–4	17 (35%)	0 (0%)	0.021*
Anemia			
All grade	45 (92%)	10 (91%)	0.92
Grade 3–4	13 (27%)	3 (27%)	0.96
Thrombocytopenia			
All grade	31 (63%)	10 (91%)	0.075
Grade 3–4	9 (18%)	0 (0%)	0.12

ESCC: esophageal squamous cell carcinoma; HNSCC: head/neck squamous cell carcinoma; CCRT: concurrent chemoradiotherapy.

^*^Statistically significant.
