# Supplementary material for: Treatment Outcomes of Patients with Locally Advanced Synchronous Esophageal and Head/Neck Squamous Cell Carcinoma Receiving Curative Concurrent Chemoradiotherapy
Source: Sci Rep. 2017 Jan 30;7:41785. doi: 10.1038/srep41785 (PMC5278381; doi:10.1038/srep41785)
Supplement: Supplementary Information [file srep41785-s1.pdf]

Title: Treatment Outcomes of Patients with Locally Advanced Synchronous Esophageal and Head/Neck Squamous Cell Carcinoma Receiving Curative Concurrent Chemoradiotherapy

Yen-Hao Chen, Hung-I Lu, Chih-Yen Chien, Chien-Ming Lo, Yu-Ming Wang, Shang-Yu Chou,

Yan-Ye Su, Li-Hsueh Shih, and Shau-Hsuan Li

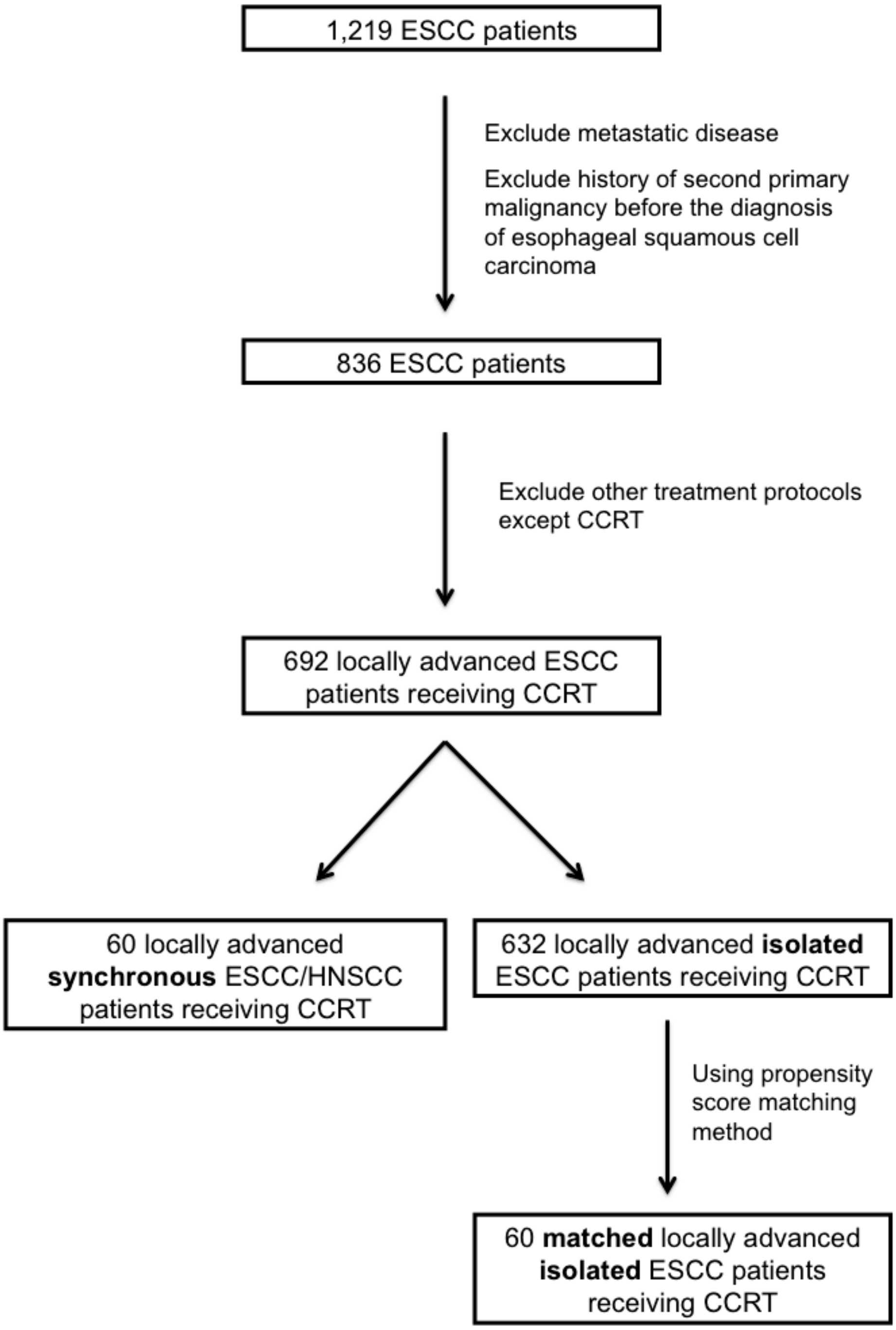

Supplement Figure S1. Algorithm for patients with locally advanced synchronous ESCC/HNSCC and patients with isolated ESCC. ESCC: esophageal squamous cell carcinoma; HNSCC: head and neck squamous cell carcinoma; CCRT: concurrent chemoradiotherapy.
